# Predicting pathway cross-talks in ankylosing spondylitis through investigating the interactions among pathways

**DOI:** 10.1590/1414-431X20176698

**Published:** 2017-11-13

**Authors:** Xiang Gu, Cong-Jian Liu, Jian-Jie Wei

**Affiliations:** 1 Department of Orthopedics, People's Hospital of Ri Zhao, Ri Zhao, Shandong, China; 2 Department of Orthopedics, Weihaiwei People's Hospital, Weihai, Shandong, China

**Keywords:** Ankylosing spondylitis, Monte Carlo cross-validation, Differentially expressed genes, Random forest classification, Pathway cross-talk

## Abstract

Given that the pathogenesis of ankylosing spondylitis (AS) remains unclear, the aim of this study was to detect the potentially functional pathway cross-talk in AS to further reveal the pathogenesis of this disease. Using microarray profile of AS and biological pathways as study objects, Monte Carlo cross-validation method was used to identify the significant pathway cross-talks. In the process of Monte Carlo cross-validation, all steps were iterated 50 times. For each run, detection of differentially expressed genes (DEGs) between two groups was conducted. The extraction of the potential disrupted pathways enriched by DEGs was then implemented. Subsequently, we established a discriminating score (DS) for each pathway pair according to the distribution of gene expression levels. After that, we utilized random forest (RF) classification model to screen out the top 10 paired pathways with the highest area under the curve (AUCs), which was computed using 10-fold cross-validation approach. After 50 bootstrap, the best pairs of pathways were identified. According to their AUC values, the pair of pathways, antigen presentation pathway and fMLP signaling in neutrophils, achieved the best AUC value of 1.000, which indicated that this pathway cross-talk could distinguish AS patients from normal subjects. Moreover, the paired pathways of SAPK/JNK signaling and mitochondrial dysfunction were involved in 5 bootstraps. Two paired pathways (antigen presentation pathway and fMLP signaling in neutrophil, as well as SAPK/JNK signaling and mitochondrial dysfunction) can accurately distinguish AS and control samples. These paired pathways may be helpful to identify patients with AS for early intervention.

## Introduction

Ankylosing spondylitis (AS) is a chronic inflammation disorder that attacks sacroiliac joints and spine (1) with a 0.3% incidence rate in the Asian population (2). AS causes severe back pain, stiffness and new bone formation, and results in progressive joint ankylosis further decreasing quality of life (3). Unfortunately, the disease condition, including disease activity, progression and prognosis, are very hard to define in AS (4). Until now, the underlying molecular processes driving the AS progress are still unclear. Consequently, investigation on the pathogenesis of AS is urgently needed.

In recent years, genetic-associated research has detected several new genes related to AS. Of note, some of these genes seem specific for AS, but others have pleiotropic associations (5,6). Moreover, these studies offer little information concerning changes in gene activity during the progression of the disease. Fortunately, gene expression profiling provides a “snapshot” of cellular activity, supplying information on molecular mechanisms mediating disease changes, and can produce diagnostic gene sets. A number of recent studies have defined transcriptional profiles generated from peripheral blood mononuclear cells for AS. GSE25101 is one of the microarray profiles of AS that was reported by Pimentel-Santos et al. (7), who identified a set of differentially expressed genes (DEGs), which were highly connected with AS, partially regulating the inflammatory process and joint destruction. In 2015, Zhao et al. (8) used the same microarray profile of GSE25101 to predict the potential AS-related genes, such as *RPL17*, *MRPL22*, *PSMA6* and *PSMA4*. In 2015, also using the same data, Shi et al. (9) identified 284 DEGs correlated with AS (such as *MYH9*, *BCL11B* and *CD4*), and detected the pathway for immune response regulation. Nevertheless, so far, most studies that assessed the genetics of AS focused on a single gene or a single pathway. However, pathway cross-talk is frequently neglected.

In general, different pathways interact with each other, and an abnormity in one pathway may influence the activities of many other related pathways. Understanding pathway interactions might be beneficial to explore the pathogenesis of AS. No reliable method is used to quantify the cross-talks for pathway pairs (10). However, integrating DEGs information and pathway information with Monte Carlo cross-validation has been proposed to quantify the cross-talk between pathways pairs (11). Monte Carlo cross-validation provided by Shao (12) has been demonstrated to decrease the risk of overfitting the model, and has been used to evaluate the prediction ability of the selected model.

Thus, in the current study, to explore the pathogenesis of AS, we undertook the microarray data analysis of AS to identify the significant pathways considering the functional dependency among pathways using Monte Carlo cross-validation. We believe that our results may contribute to a better understanding of the molecular processes driving AS progression.

## Material and Methods

**Figure 1.**
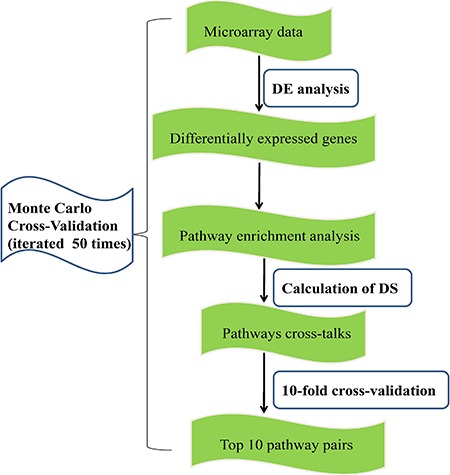
Flowchart displaying a brief overview of the main protocol. DE: differential expression; DS: discriminating score.

Using microarray profile as well as biological pathways as study objects, Monte Carlo cross-validation method was used to identify the significant pathways. In this process, all steps were iterated 50 times. For each run, we implemented differential expression analysis (DEA), pathways enrichment analysis, calculation of a discriminating score (DS), and random forest (RF) classification. After 50 runs, the top 10 pathway pairs with the best area under the curve (AUC) values were identified (significant paired pathways). The flow chart of the analysis is shown in Figure 1.

### Microarray data

The expression profile of GSE25101 deposited by Pimentel-Santos et al. (7) was downloaded from Gene Expression Omnibus (http://www.ncbi.nlm.nih.gov/geo/), based on the GPL6947 platform of Illumina Human HT-12 V3.0 expression BeadChips. In the GSE25101, there were 16 active AS patients and 16 normal controls with matched gender and age. Included AS patients had Bath Ankylosing Spondylitis Disease Activity Index (BASDAI) scores >4 and Bath Ankylosing Spondylitis Functional Index (BASFI) scores >4. The raw probe annotation files were obtained for subsequent analysis.

### Differential expression analysis

The raw probe sets were pre-processed by means of robust multiarray averaging method. Then, the probes were mapped to the genomics, and the human gene symbols were obtained. Afterwards, Bayesian approach (13) was utilized to detect DEGs. With the goal of avoiding the multiple testing bias, which might cause false positive results, the Benjamini-Hochberg method (14) was used for multiple correction, and false discovery rate (FDR) distribution for each gene was obtained. Eventually, |log2FC| >1 and FDR <0.05 were set as the cut-off criteria to extract DEGs between AS and control samples.

### Pathway enrichment analysis

To extract a cluster of pathways significantly enriched by DEGs, pathway enrichment analysis from DEGs was conducted. First, a total of 589 biological pathways were derived from the Ingenuity Pathways Analysis (IPA) software (http://www.ingenuity.com/) (15). Then, we assessed the enrichment effect based on Fisher's exact test, aiming to place DEGs in an IPA pathway and to screen out the pathways responsible for coordinating their activity. The pathways with P<0.01 were identified. Afterwards, the raw P values were corrected for multiple testing through Benjamini-Hochberg procedure (14). In the current study, the pathways with FDR <0.05 were considered as differential pathways.

### DS for pathway cross-talk

The DS is often used to compare the expression levels in the samples with amplification and the samples without amplification (16). The DS indicates the relationships between pathway pairs. Therefore, we calculated the DS to further analyze the pathway cross-talks in our study. In detail, we computed a DS by comparing the expression levels of each pathway pair involved in DEGs in each sample based on the formula used in a previous study by Orsetti et al. (16). A larger DS denotes a higher difference in activity of the pairs.

### Selecting the best pathway pairs

RF is a powerful classifier utilized to handle two issues of variable selection, and it has become a standard analysis tool in bioinformatics (17). Thus, in the present work, we employed a RF model on the pathway pairs to estimate the classification performance of this method according to AUC index. The 10-fold cross-validation method was utilized to calculate AUC based on mtry and ntree parameters. The ‘mtry', is the number of variables randomly sampled as candidates at each split, and is equal to sqrt(p), where p is the variable count in the data matrix. The ‘ntree', is the count of trees grown, amounted to 500. Subsequently, we sorted all AUC values in descending order, and the top 10 pathway pairs were extracted.

As reported, during the validation analysis, the ratio of 6 to 4 is a commonly used distribution proportion (18). Thus, the Monte Carlo cross-validation method randomly assigned 60% of the original microarray profile to make up for the training set and assigned the remaining 40% to form the testing data. This procedure was iterated 50 times, randomly producing new training and testing sets each time. For each run, we used a training set to extract DEGs, the pathways enriched by DEGs, and the DS values for the top 10 pathway pairs with the best AUCs between AS and control groups; we utilized the testing data to validate the top 10 pathway pairs. At the end of the 50 runs, the top 10 pathway pairs sorted in descending rank were extracted in 50 bootstraps. Eventually, the list of the top 10 pathway pairs ranked for all 50 bootstraps were considered as the significant ones.

## Results

### DEGs identification and pathway enrichment analysis

After robust microarray analysis, 11,586 genes were identified for differential expression analysis. According to |log2FC| >1 and FDR < 0.05, 19 genes were found to be differentially expressed between AS and control samples, as listed in Table 1. Moreover, 46 significant pathways enriched by DEGs were detected. Table 2 displays the names of significant pathways enriched from DEGs, the corresponding FDR values, count of genes for each pathway, and count of common genes between DEGs and genes in the pathways. Then, we computed a DS via comparing the expression levels of each pair of pathways involved in DEGs in each sample. The distribution of DS values is shown in Supplementary Table S1. The DS values of 4 pathway cross-talks were higher than 0.1, which included the role of NFAT in regulation of the immune response/prostanoid biosynthesis (DS=0.212938), the role of NFAT in regulation of the immune response/eicosanoid signaling (DS=0.212938), the role of NFAT in regulation of the immune response/EIF2 signaling (DS=0.182708), and the role of NFAT in regulation of the immune response/activation of IRF by cytosolic pattern recognition receptors (DS=0.111934).

**Table 1.** List of differentially expressed genes (DEGs).

Genes	Log false change	False discovery rate
*IL2RB2*	–1.055680	0.000366
*GNG1131*	1.452149	0.000369
*ADGRG16*	–1.214451	0.000772
*S100A1225*	1.438137	0.001421
*KIR2DL31*	–1.046953	0.001876
*TMA728*	1.121291	0.002057
*CHMP513*	1.014122	0.002439
*PRF11*	–1.018001	0.002573
*LSM322*	1.051145	0.003032
*PPBP4*	1.233433	0.003703
*UQCRBP123*	1.154782	0.004073
*COX7B25*	1.174587	0.005779
*RPL2325*	1.071111	0.012291
*MCEMP17*	1.078964	0.012743
*LY9627*	1.206473	0.022822
*PTGDS9*	–1.115798	0.023302
*COMMD628*	1.108966	0.027617
*RAP1GAP18*	1.336118	0.037313
*DEFA45*	1.013721	0.049562

**Table 2.** Pathways enriched by differentially expressed genes (DEGs).

Pathway	Gene in pathway	FDR	No. of common gene
Role of NFAT in regulation of the immune response	160	0.000919	2
Prostanoid biosynthesis	9	0.002692	1
G protein signaling mediated by tubby	31	0.009248	1
Graft-versus-host disease signaling	39	0.009842	1
Antigen presentation pathway	34	0.010139	1
Glutamate receptor signaling	56	0.016358	1
Activation of IRF by cytosolic pattern recognition receptors	60	0.017539	1
Eicosanoid signaling	60	0.017834	1
Antiproliferative role of somatostatin receptor 2	60	0.017834	1
CCR5 signaling in macrophages	62	0.018129	1
T helper cell differentiation	62	0.018424	1
IL-4 signaling	70	0.020781	1
Altered T cell and B cell signaling in rheumatoid arthritis	76	0.021075	1
Ephrin B signaling	73	0.021663	1
Crosstalk between dendritic cells and natural killer cells	84	0.024013	1
Adrenergic signaling	85	0.024893	1
G beta gamma signaling	88	0.026066	1
SAPK/JNK signaling	88	0.026066	1
IL-1 signaling	91	0.026944	1
iCOS-iCOSL signaling in T helper cells	97	0.028407	1
Type I diabetes mellitus signaling	101	0.029868	1
fMLP signaling in neutrophils	106	0.031035	1
G_s signaling	108	0.031327	1
CD28 signaling in T helper cells	107	0.031327	1
Androgen signaling	110	0.031618	1
PKC_ signaling in T lymphocytes	107	0.031618	1
CCR3 signaling in eosinophils	112	0.032784	1
P2Y purigenic receptor signaling pathway	118	0.034821	1
G_i signaling	120	0.035112	1
Cardiac _-adrenergic signaling	132	0.038884	1
Relaxin signaling	132	0.038884	1
G_q signaling	143	0.041778	1
Tec kinase signaling	149	0.041799	1
CXCR4 signaling	151	0.042376	1
Dendritic cell maturation	159	0.042953	1
EIF2 signaling	171	0.043119	1
CREB signaling in neurons	169	0.043452	1
RhoGDI signaling	172	0.043842	1
Ephrin receptor signaling	172	0.043916	1
Role of NFAT in cardiac Hypertrophy	174	0.044702	1
IL-8 signaling	183	0.044828	1
Breast cancer regulation by stathmin1	190	0.044924	1
Thrombin signaling	187	0.045471	1
Huntington's disease signaling	215	0.047115	1
Cardiac hypertrophy signaling	217	0.047967	1
Phospholipase C signaling	219	0.049534	1

Common gene: the overlap between DEGs and genes in the pathway. FDR: false discovery rate.

### Identifying the best pathway pairs

With the goal of assessing the classification ability of this approach, we used 10-fold cross-validation to calculate the AUC values for pathway pairs using the RF model. Each pathway pair was then ranked based on its corresponding AUC value. Of note, there were 35 pairs of pathways with AUC not less than 0.800. It is known through the literature that AUC >0.7 is determined as good, and an AUC of 1.0 suggests a perfect classification (19). Greater AUCs indicate better disease classification, that is, a stronger correlation between the pathways and the given disease. Hence, to better understand the molecular mechanisms of AS, we focused more in the top 10 pathway pairs, as reported by Colaprico et al. (11). Table 3 demonstrates the top 10 paired pathways with the best classification performance for AS and control samples for all 50 runs. The pair ‘antigen presentation pathway’ and ‘fMLP signaling in neutrophils’ had the best AUC value of 1.0. Moreover, the pair ‘activation of IRF by cytosolic pattern recognition receptors’ and glucocorticoid receptor signaling’ revealed a good classification ability with a 0.995 AUC. Similar performance was observed in the pair ‘T helper cell differentiation’ and ‘CXCR4 signaling’ with AUC of 0.992.

**Table 3.** Top 10 pathway pairs with the highest AUC values.

Pairs of pathways	AUC
(1a) Antigen presentation pathway (1b) fMLP signaling in neutrophils	1.000
(2a) Activation of IRF by cytosolic pattern recognition receptors (2b) Glucocorticoid receptor signaling	0.995
(3a) T helper cell differentiation (3b) CXCR4 signaling	0.992
(4a) Altered T cell and B cell signaling in rheumatoid arthritis (4b) Colorectal cancer metastasis signaling	0.988
(5a) Type I diabetes mellitus signaling (5b) G_s signaling	0.988
(6a) Type I diabetes mellitus signaling (6b) Relaxin signaling	0.988
(7a) fMLP signaling in neutrophils (7b) CD28 signaling in T helper cells	0.986
(8a) CD28 signaling in T helper cells (8b) Androgen signaling	0.982
(9a) PKC_ signaling in T lymphocytes (9b) Relaxin signaling	0.879
(10a) Eicosanoid signaling (10b) Granulocyte adhesion and diapedesis	0.873

AUC: area under the curve.

After this, the top 10 pairs of pathways were extracted based on the occurrence frequency in the 50 bootstraps ≥3. According to this procedure, the pair ‘SAPK/JNK signaling’ and ‘mitochondrial dysfunction’ were involved in 5 bootstraps, the pair ‘mitochondrial dysfunction’ and ‘G beta gamma signaling’ appeared in 4 runs, and the pair ‘mitochondrial dysfunction’ and ‘G protein signaling mediated by Tubby’ also appeared in 4 runs. Importantly, among these top 10 pathway pairs, the ‘mitochondrial dysfunction’ interacted with 6 different pathways. Specific information is shown in Table 4.

**Table 4.** Top 10 pathway pairs with occurrence number not less than 3.

Pathway pairs	Total occurrence number
(1a) EIF2 signaling (1b) LXR/RXR activation	3
(2a) Glutamate receptor signaling (2b) Mitochondrial dysfunction	3
(3a) EIF2 signaling (3b) iNOS signaling	3
(4a) IL-1 signaling (4b) Mitochondrial dysfunction	3
(5a) CCR5 signaling in macrophages (5b) Mitochondrial dysfunction;	3
(6a) EIF2 signaling (6b) Hepatic fibrosis/hepatic stellate cell activation	3
(7a) EIF2 signaling (7b) MIF regulation of innate immunity	3
(8a) G protein signaling mediated by Tubby (8b) Mitochondrial dysfunction	4
(9a) G beta gamma signaling; (9b) Mitochondrial dysfunction	4
(10a) SAPK/JNK signaling (10b) Mitochondrial dysfunction	5

## Discussion

AS, as a common rheumatic disorder, leads to inflammatory back pain, thereby reducing the quality of life (20). The potential molecular mechanism of AS remains unclear. In recent years, gene expression profiles have been widely used to identify disease-related biomarkers (21,22), several of them having similar functions, however reproducibility is poor. In this condition, these biomarkers may not have precise classification ability. With the goal of solving this challenge, extraction of biological pathways involved in a given phenotype is a key process. Pathway-based bio-signatures are more reproducible and frequently obtain better classification ability than single gene biomarkers (19). However, currently, most approaches regard the pathways to be independent, not considering the interactions between them, called “cross-talk” (23). The cross-talks among pathways indicate the regulatory interaction among different pathways. Of note, detection of cross-talks among pathways better reveal the pathway functions and contribute more to the understanding of the synergistic effects on cellular processes, compared with individual pathways (24). Furthermore, several reports demonstrated the potential function of pathway cross-talks in therapeutic strategies (25,26). Although there are many merits of pathway cross-talk in disease treatment, pathways amount of cross-talk interactions have not been completely studied. Most importantly, no available technique can quantify the cross-talks for pathway pairs (10). Integrating DEGs information and the pathway information with Monte Carlo cross-validation has been proposed to quantify the cross-talk between pathways pairs (11). Consequently, in the present work, Monte Carlo cross-validation analysis was employed to uncover the best paired pathways that could distinguish between AS and control samples. We found 35 paired pathways with AUCs not less than 0.800, after evaluating the top 10 paired pathways. Thus, the pathogenesis of AS may be related with the expression alterations of these paired pathways.

The pathway pair ‘antigen presentation pathway’ and ‘fMLP signaling in neutrophils’ got the best AUC value of 1.000, which indicated that this pathway cross-talk could distinguish AS patients from the normal subjects. As reported, exogenous antigens are presented by major histocompatibility complex (MHC) class I molecules (27). Significantly, MHC class I molecules have been suggested to play important roles in immune surveillance by binding to CD8+ T cells, which act in concert towards antigen processing as well as antigen presentation machinery (28,29). HLA-27 and ERAP1 are central members in the antigen presentation machinery, which have been shown to contribute to the AS risk (30). HLA-27 can regulate the migration of neutrophil and neutrophils exert key functions in the innate immune response (31). A previous study published by Biasi et al. exhibited an increased response to fMLP by circulating neutrophils in AS patients (31). Accordingly, the cross-talk between antigen presentation pathway and fMLP signaling in neutrophils might be strongly correlated with the etiology of AS, probably via regulating the immune response.

Bone formation (for example, syndesmophytes) is a common feature of AS (32). Furthermore, bone formation and development depend on the balance between osteoblasts-mediated bone formation and osteoclasts-induced bone resorption, and this bone homeostasis is disrupted in an inflammation environment (33,34). TNF-*α*, as a key pro-inflammatory cytokine, is responsible for the inflammation-related bone loss, and TNF-*α* can suppress BMP-mediated osteoblastogenesis through activating the SAPK/JNK pathway (35). Furthermore, endoplasmic reticulum (ER) stress can provide the links with the inflammatory responses, and result in the activation of JNK by reactive oxygen species (ROS) (36). Furthermore, mitochondria can contribute to the production of ROS (37). As documented, ROS attacks are directed primarily towards the polyunsaturated fatty acids of the membrane lipids, inducing lipid peroxidation, which further results in the disorganization of cell structure and function (38). Additionally, ROS have been indicated to be possible mediators of tissue damage, which is related to AS (39). Therefore, we infer that the alteration of the pathway pair ‘SAPK/JNK signaling’ and ‘mitochondrial dysfunction’ might induce AS onset and progression by affecting inflammatory and oxidative metabolism as mentioned above.

Nevertheless, several study limitations must be noted. First, the sample size was rather small. Second, this was a preliminary study of mechanisms underlying AS and results were achieved based on *in silico* analysis without validation in animal models or patient tissues. Thus, these pathway pairs should be further investigated using western blotting or PCR-based experiments to reveal the pathway changes in AS.

In conclusion, our analysis provided new knowledge for AS and identified several bio-signatures for this disease. Based on our results, the detected pathway cross-talks might be helpful to identify patients with AS for early intervention. However, these paired pathways call for future functional studies.

## Supplementary material

Click here to view [pdf].
